# Statistical power for MACE and individual secondary endpoints in cardiovascular outcomes trials for type 2 diabetes: a systematic review

**DOI:** 10.1038/s41598-022-25296-x

**Published:** 2022-12-06

**Authors:** Sebastian Birker, Juris J. Meier, Michael A. Nauck

**Affiliations:** 1 Diabetes, Endocrinology, Metabolism Section, Medical Department I, Katholisches Klinikum Bochum gGmbH, St. Josef Hospital, Ruhr-University Bochum, Gudrunstr. 56, 44791 Bochum, Germany; 2 Department of Internal Medicine, Augusta Hospital, Bochum, Germany

**Keywords:** Cardiology, Endocrinology, Medical research

## Abstract

Cardiovascular outcomes trials (CVOTs) with novel drugs to treat type 2 diabetes have uniformly chosen the composite “major adverse cardiovascular events (MACE)” as their primary endpoint, but they also report hazard ratios for individual cardiovascular outcomes (myocardial infarction, stroke, cardiovascular death, all-cause death, hospitalization for heart failure). We wanted to scrutinize the power to identify significant differences with respect to individual as compared to composite outcomes. We estimated post hoc the statistical power to detect significant differences of 10–25% for published studies, comparing the proportions of patients with an event (two-sided log-rank tests). For MACE, the power to detect a 15% difference ranged from 82.3 to 100.0% for larger trials, but was only 69.1 and 50.5 for smaller, preliminary trials (SUSTAIN-6 and PIONEER-6). For individual endpoints, the power, as a rule, was substantially lower. In conclusion, cardiovascular outcomes trials had appropriate power to detect significant reductions in hazard ratios with respect to the primary endpoint, but not for individual cardiovascular outcomes. This was particularly the case for small, preliminary studies. Our results call for caution when comparing results regarding individual endpoints between CVOTs, if the aim is to identify heterogeneity within or between medication classes.

## Introduction

Cardiovascular outcomes trials employing novel diabetes drugs (e.g., SGLT-2 inhibitors^1–4^, DPP-4 inhibitors^5–8^, and GLP-1 receptor agonists^9–16^) have been published between 2013 and today. These studies have established SGLT-2 inhibitors and GLP-1 receptor agonists as the two medication classes for the treatment of type 2 diabetes, which do not only provide glycaemic control and body weight reductions without provoking hypoglycaemic episodes, but also show a potential for reducing the risk for a composite endpoint composed of cardiovascular events like acute myocardial infarction, stroke, cardiovascular death (major adverse cardiovascular events or MACE) in the majority of studies^1–3,10,11,13,14,16^. In addition, SGLT-2 inhibitors (SGLT-2 Is) also reduce the vents of hospitalization for congestive heart failure^1–4^, and SGLT-2 Is, more than GLP-1 RAs, prevent renal endpoints^2–4,11,16–20^.

While meta-analyses have confirmed these general patterns of effects for SGLT-2 inhibitors ^21–23^ and GLP-1 RAs^24^, and, thus, have established “class effects”, on first sight, i.e., in the absence of head-to-head trials, some heterogeneity is obvious for results obtained with various SGLT-2 Is (effects on MACE reduction vs. placebo significant^1–3^ or not^4^), DPP-4 inhibitors (worsening of the risk for hospitalization because of congestive heart failure with saxagliptin^5,25^, and, as a trend, alogliptin^6,26^ but not with sitagliptin^7^ or linagliptin^8^) and, in particular, for GLP-1 RAs (not even a trend for improved MACE outcomes for lixisenatide^9^, highly significant reductions in MACE events with liraglutide^10^, semaglutide s.c.^11^, albiglutide^13^, dulaglutide^14^, and efpeglenatide^16^). The latter findings are well compatible with significant differences regarding pharmacokinetic behaviour and the selection of dosages for the obviously heterogeneous class of GLP-1 RAs^27,28^.

**Figure 1:**
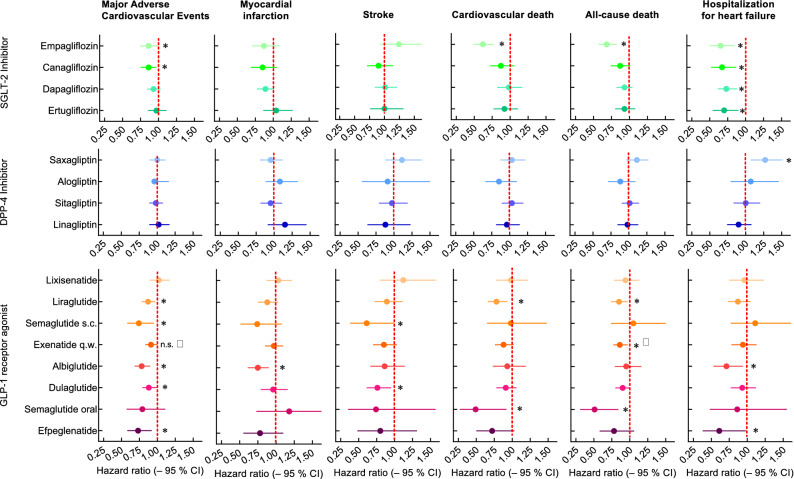
Hazard ratios (± 95% confidence intervals) for major cardiovascular adverse events (MACE) as well as its components ([non-fatal] myocardial infarction and stroke, cardiovascular death) and all-cause death and hospitalization for congestive heart failure reported in cardiovascular outcomes studies comparing novel glucose-lowering medications with placebo treatment (both on a background of standard of care) in patients with type 2 diabetes. Asterisks indicate a significant difference to placebo treatment; †: *p* = 0.06; ‡: This nominally significant difference cannot claim significance due to hierarchical testing (difference in MACE not significant).

Current guidelines and treatment recommendations suggest to consider medications, which can provide evidence for cardiovascular benefits for those at risk for such events^29–31^. This concerns subjects with pre-existing atherosclerotic cardiovascular disease as well as patients with chronic kidney failure (typically associated with a high cardiovascular risk), and those with congestive heart failure^29–31^. Following this recommendation should prompt a comparison of reported cardiovascular outcomes between trials reporting beneficial effects in this respect, i.e., within the classes of SGLT-2 Is and GLP-1 RAs, based on the heterogeneity of results within one class of glucose-lowering medications (Fig. 1). A superficial inspection of such data may suggest that albiglutide is particularly suited to reduce acute myocardial infarction events^13^, while semaglutide (for once-weekly subcutaneous injection)^11^ or dulaglutide^14,32^ are particularly suited to prevent stroke events. Along the same lines, liraglutide^10^ and oral semaglutide^15^ may appear to be the drugs of choice to prevent cardiovascular or all-cause death. We have to be aware that such comparisons are indirect based on the general design of such trials as placebo-controlled trials on a background of the standard of care. One important prerequisite for meaningful comparisons is a sufficient power provided by reported trials to detect significant differences between active and placebo treatment. It is the purpose of the present analysis to provide post hoc estimates of the power to detect differences between 10 and 25°% (corresponding to the magnitude of relative changes observed in relevant cardiovascular outcomes trials^1–16^). Our estimates will provide important background information for the interpretation of such trials, in particular regarding the comparison between different classes (e.g., SGLT-2 Is and GLP-1 RAs) or within these classes, in order to identify medications that will best help individual patients to address their cardiovascular risk.

## Results

### Identification of suitable publications

Sixteen studies were identified as suitable for the present analysis^1–16^). Four studies each reported results from cardiovascular outcomes trials with SGLT-2 Is^1–4^ or DPP-4 Is^5–8^, respectively. Eight studies concerned treatment with various GLP-1 RAs^9–16^). Study and patient characteristics at baseline are compiled as Supplementary Tables 1 and 2, respectively. Table 1 compiles the most relevant information on study size (number of patients participating, study duration, and overall patient years of observation) and event numbers and the proportion of patients reporting a MACE event. It is obvious, that two studies (SUSTAIN-6^11^ and PIONEER-6^15^, examining semaglutide s.c. once weekly, and oral semaglutide administered daily, respectively) are much smaller in size (patient numbers, observation period, patient years of observation) as compared to all other studies (however, with considerable heterogeneity within the latter category). The reason is that the study objectives for SUSTAIN-6 and PIONEER-6 were different: Both were designed to provide preliminary evidence for safety pre-approval (with the necessity for additional post-approval studies in mind). Since the criteria for preliminary cardiovascular safety are more relaxed (hazard ratio for the MACE composite endpoint with upper 95% confidence interval < 1.80) than for the definite proof of cardiovascular safety (hazard ratio for the MACE composite endpoint with upper 95% confidence interval < 1.30) according to guidance by the food and drug administration of the United States (FDA) (https://www.fda.gov/media/71297/download), these criteria can be satisfied with fewer MACE events, allowing for fewer patients and a shorter observation period.

**Table 1:** Sample size (total study population), median study duration, patient years of observation, and number of events reported with placebo or active drug treatment for major adverse cardiovascular events (MACE), all-cause deaths, and hospitalization for heart failure.

Class	Study acronym	Study population (n)	Study duration [years]	Patient years of observation	Major adverse cardiovascular events		All-cause deaths		Hospitalization for congestive heart failure	
					Placebo treatment	Active drug treatment	Placebo treatment	Active drug treatment	Placebo treatment	Active drug treatment
					Events [n] (proportion [%])	Events [n] (proportion [%])	Events [n] (proportion [%])	Events [n] (proportion [%])	Events [n] (proportion [%])	Events [n] (proportion [%])
SGLT-2 inhibitors	EMPAREG-OUTCOME	7020	3.2	20,514	282 (12.1)	490 (10.5)	194 (8.3)	269 (5.7)	95 (4.1)	126 (2.7)
	CANVAS	10,142	2.4	35,968	485 (11.2)	554 (9.6)	300 (6.9)	356 (6.1)	134 (3.1)	113 (2.0)
	DECLARE-TIMI 58	17,160	4.2	69,547	803 (9.4)	756 (8.8)	570 (6.6)	529 (6.2)	286 (3.3)	212 (2.5)
	VERTIS-CV	8246	3.0	27,046	327 (11.9)	653 (11.9)	254 (9.2)	473 (8.6)	99 (3.6)	139 (2.5)
DPP4 inhibitors	SAVOR TIMI 53	16,492	2.1	33,645	609 (7.2)	613 (7.3)	378 (4.2)	420 (4.9)	228 (2.8)	289 (3.9)
	EXAMINE	5380	1.5	7608	316 (11.8)	305 (11.3)	173 (6.5)	153 (5.7)	79 (2.9)	85 (3.1)
	TECOS	14,671	3.0	43,536	746 (10.2)	745 (10.2)	537 (7.3)	547 (7.5)	229 (3.1)	228 (3.1)
	CARMELINA	6979	2.2	13,352	420 (12.1)	434 (12.4)	373 (10.7)	367 (15.5)	226 (6.5)	209 (6.0)
GLP-1 receptor agonists	ELIXA	6068	2.1	12,418	289 (12.8)	397 (13.1)	223 (7.4)	211 (7.0)	127 (4.2)	122 (4.0)
	LEADER	9340	3.8	34,926	694 (14.9)	608 (13.0)	447 (9.6)	381 (8.2)	248 (5.3)	218 (4.7)
	SUSTAIN-6	3297	2.1	6855	146 (8.9)	108 (6.6)	60 (3.6)	62 (3.8)	54 (3.3)	59 (3.6)
	EXSCEL	14,752	3.2	46,302	905 (12.2)	839 (11.4)	584 (7.9)	507 (6.9)	231 (3.1)	219 (3.0)
	HARMONY OUTCOMES	9463	1.6	14,927	428 (9.0)	338 (7.0)	205 (4.0)	196 (4.0)	88 (1.9)	66 (1.4)
	REWIND	9901	5.4	52,680	663 (13.4)	594 (12.0)	592 (12.0)	536 (10.8)	226 (4.6)	213 (4.3)
	PIONEER 6	3183	1.3	4210	76 (4.8)	61 (3.8)	45 (2.8)	23 (1.4)	24 (1.5)	21 (1.3)
	AMPLITUDE-O	4076	1.8	7395	125 (9.2)	189 (7.0)	69 (4.9)	111 (4.1)	31 (2.3)	40 (1.5)

### Power to detect differences in MACE between active drug and placebo treatment with the number of endpoints observed in individual studies

**Figure 2:**
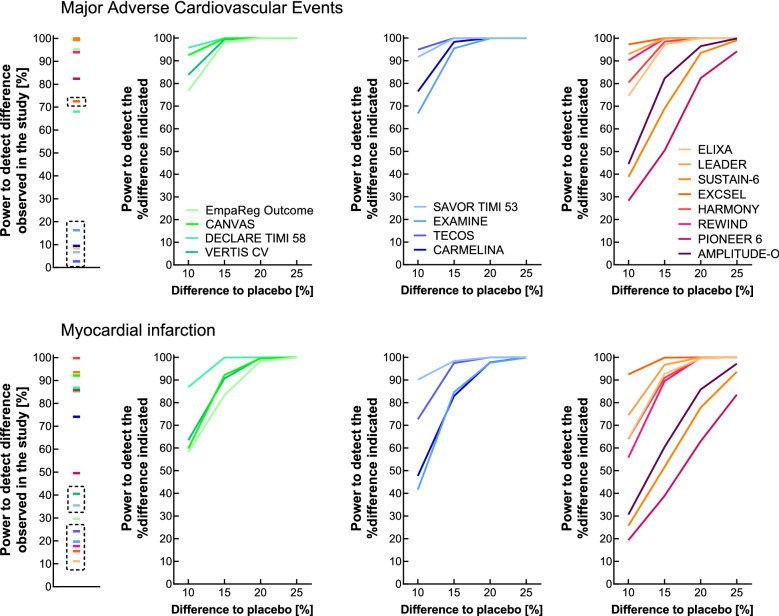
Post hoc power estimation of for cardiovascular outcomes concerning trials comparing novel glucose-lowering medications with placebo treatment (both on a background of standard of care) in patients with type 2 diabetes for major adverse cardiovascular outcomes (MACE; upper row of panels) and non-fatal myocardial infarction (lower row of panels). Left hand panels display the power calculated regarding the hazard ratio as reported in each individual study. The second through fourth columns of panels illustrate the power to detect differences by 10, 15, 20, or 25% versus the proportion of patients with events reported with placebo treatment with SGLT-2 inhibitors (second column of panels), DPP-4 inhibitors (third column of panels) and GLP-1 receptor agonists (fourth column of panels). The power estimates for study results indicating a negligible difference to placebo treatment (≤ 10%) are highlighted with a dashed rectangle, because small differences are typically associated with low power.

Significant differences in the number of MACE events between active drug and placebo treatment were detected in all but one studies with SGLT-2 Is (exception: VERTIS-CV trial using ertugliflozin), and in all but one publication on GLP-1 RAs (exception: ELIXA, examining lixisenatide effects), and in none of the DPP-4 inhibitor trials (Fig. 1). In those trials reporting significant reductions in the risk for MACE (Fig. 1), the power to detect this difference (ranging from 13 to 27% reduction in MACE) was 68.1 (DECLARE-TIMI 58; dapagliflozin) to 99.7 (AMPLITUDE-O; efpeglenatide; Fig. 2). 2 out of 4 SGLT-2 inhibitor trials, none of the DPP-4 I trials, and 6 out of 8 GLP-1 RA trials had an estimated power of > 80% to detect significant differences between active drug and placebo treatment as they were described in those studies (Fig. 2). Trials not describing a significant difference between active drug and placebo treatment typically showed small effect sizes (Fig. 1) associated with a low post hoc power estimate (Fig. 2).

### Power to detect differences in individual cardiovascular endpoints between active drug and placebo treatment with the number of endpoints observed in individual studies

**Figure 3:**
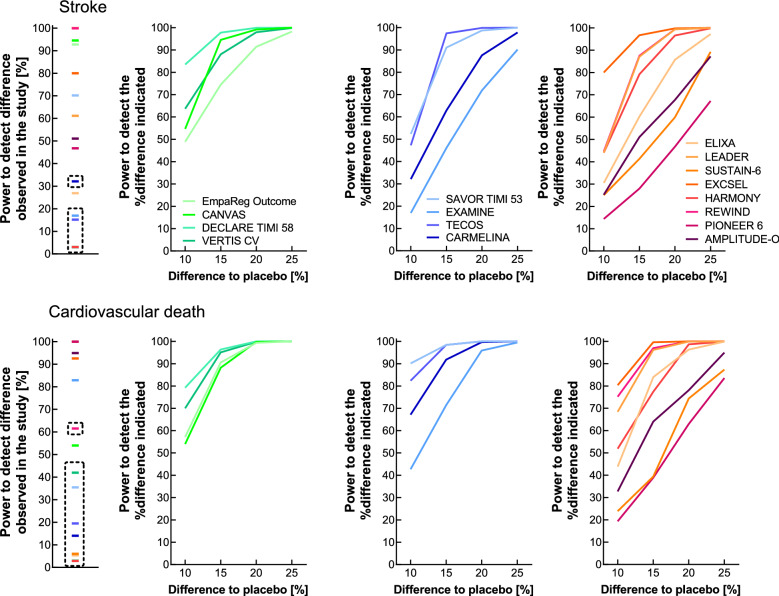
Post hoc power estimation of for cardiovascular outcomes concerning trials comparing novel glucose-lowering medications with placebo treatment (both on a background of standard of care) in patients with type 2 diabetes for non-fatal stroke (upper row of panels) and cardiovascular death (lower row of panels). Left hand panels display the power calculated regarding the hazard ratio as reported in each individual study. The second through fourth columns of panels illustrate the power to detect differences by 10, 15, 20, or 25% versus the proportion of patients with events reported with placebo treatment with SGLT-2 inhibitors (second column of panels), DPP-4 inhibitors (third column of panels) and GLP-1 receptor agonists (fourth column of panels). The power estimates for study results indicating a negligible difference to placebo treatment (≤ 10%) are highlighted with a dashed rectangle, because small differences are typically associated with low power.

A sufficient power (> 80%) to detect significant differences, as they occurred in individual trials between active drug and placebo treatment, with respect to (non-fatal) myocardial infarction was estimated in 2 out of 4 SGLT-2 I studies, in none of the DPP-4 I trials, and in 4 out of 8 GLP-1 RA studies (Fig. 2). The respective numbers concerning (non-fatal) stroke were 2 out of 4 SGLT- I studies, none out of the 4 DPP-4 I trials, and 3 out of 8 GLP-1 RA trials (Fig. 3). For cardiovascular death, this criterion was fulfilled in 1 SGLT-2 I trial and 4 GLP-1 RA studies (Fig. 3). Regarding all-cause death, the figures were 2 (out of 4 SGLT-2 I trials), 1 (out of 4 DPP-4 I studies), and 4 (out of 8 GLP-1 RA studies; Supplementary Fig. 1). All SGLT-2 inhibitor studies were sufficiently powered to detect significant differences in hospitalization for congestive heart failure. This was the case in only 1 (out of 4) and 2 (out of 8) studies reporting results on DPP-4 Is or GLP-1 RAs, respectively (Supplementary Fig. 1). Thus, for the individual CV endpoints, sufficient power was estimated for a much smaller number of trials as compared to the composite MACE endpoint. Accordingly, fewer comparisons were significant for these individual endpoints (Fig. 1).

### Estimates of the power to detect significant differences of pre-defined magnitude (10–25%) between active drug and placebo treatment

Generally speaking, as expected, the estimated power to detect significant differences between active drug and placebo treatment rose considerably with the target difference increasing from 10 to 25% (Figs. 2 and 3, Supplementary Fig. 1). As displayed in Fig. 2, the power to detect differences by > 15% between active drug and placebo treatment in MACE endpoints was high (> 80%) for all SGLT-2 I and DPP-4 I trials, and for 5 out of 8 GLP-1 RA studies (Fig. 2). Notably, the AMPLITUDE-O trial (efpeglenatide) and SUSTAIN-6 (semaglutide s.c.) as well as PIONEER-6 (oral semaglutide, both designed to provide preliminary proof of safety), had a low power even for their primary endpoint MACE, unless the target difference was higher than 20% between placebo and active drug treatment (Fig. 2).

For (non-fatal) myocardial infarction, all SGLT-2 I and DPP-4 I studies provided an estimated power > 80% to detect significances differences between active drug and placebo treatment amounting to > 15% (Fig. 2). This was also the case in 5 out of 8 GLP-1 RA trials. A lower power was estimated for the AMPLITUDE-O, SUSTAIN-6, and PIONEER-6 trials, which only had sufficient power to detect differences ≥ 20 (AMPLITUDE-O) or ≥ 25% (SUSTAIN-6 and PIONEER-6 trials, Fig. 2).

For (non-fatal) stroke, 3 out of 4 SGLT-2 I and 2 out of 4 DPP-4 I studies provided an estimated power > 80% to detect significances differences between active drug and placebo treatment amounting to > 15% (Fig. 3). This was also the case in 2 out of 8 GLP-1 RA trials (Fig. 3). The majority of GLP-1 RA trials (7 out of 8) was, however, powered to detect differences approaching 25% (Fig. 3).

For cardiovascular death, all SGLT-2 I and 3 out of 4 DPP-4 I studies provided an estimated power > 80% to detect significances differences between active drug and placebo treatment amounting to > 15%. This was also the case in 4 out of 8 GLP-1 RA trials (Fig. 3). All but one study (PIONEER-6) had the estimated power to detect differences ≥ 25% (Fig. 3).

Regarding all-cause death, all SGLT-2 I and DPP-4 I studies provided an estimated power > 80% to detect significances differences between active drug and placebo treatment amounting to > 15% (Supplementary Fig. 1). This was also the case in 5 out of 8 GLP-1 RA trials. A lower power was estimated for the AMPLITUDE-O, SUSTAIN-6, and PIONEER-6 trials, which only had 80% power to detect differences ≥ 20 (AMPLITUDE-O) or ≥ 25% (Supplementary Fig. 1), respectively.

For hospitalization because of congestive heart failure, 2 out of 4 SGLT-2 I and 3 out of 4 DPP-4 I studies provided an estimated power > 80% to detect significances differences between active drug and placebo treatment amounting to > 15% (Supplementary Fig. 1). This was also the case in 4 out of 8 GLP-1 RA trials (Supplementary Fig. 1). 5 out of 8 trials had sufficient power to detect a difference of ≥ 20%, 7 out of 8 studies provided a power of > 80% to detect a ≥ 25% difference, but one study (PIONEER-6) was not even powered to detect a difference of 25% (Supplementary Fig. 1).

### Relationship of study size with confidence intervals for hazard ratios comparing active drug vs. placebo treatment

Since confidence intervals spanning the line of unity are pivotal in determining the significance of differences between active drug and placebo treatment, we assessed the association of the width of the confidence intervals and parameters representing study size (either overall patient years of observation or numbers of events). Such an analysis is shown in Supplementary Fig. 2 for major adverse cardiovascular events (MACE). Regression analysis indicates a highly significant association (Supplementary Fig. 2), thus confirming an important role of study size (patient number) and duration, and, as a consequence, patient years of observations and (MACE) event rates, as determinants of statistical power, i.e. a pivotal prerequisite for detecting significant differences between active drug and placebo treatment.

## Discussion

Our analysis provides evidence that the cardiovascular outcomes trials examining novel diabetes medications (SGLT-2 Is^1–4^, DPP-4 Is^5–8^, and GLP-1 RAs^9–16^) were well powered to detect significant differences with respect to the primary endpoint (major adverse cardiovascular events) in the range that really occurred as the result of such clinical studies (15–25%; Fig. 2). However, for individual endpoints, this was not uniformly the case. For acute myocardial infarction, stroke, cardiovascular death, all-cause death, and hospitalization for congestive heart failure, the estimated power was lower than for the main (composite) endpoint, explained by the lower numbers of events in these categories (Table 1, Supplementary Table 3).

To judge the consequences of the relatively lower power to detect individual cardiovascular endpoints, the true differences as found on the trials have to be taken into account: Regarding MACE, 4 out of 8 GLP-1 RA trials described a reduction by 21–27% (PIONEER-6^15^, HARMONY Outcomes^13^, SUSTAIN-6^11^, and AMPLITUDE-O^16^). Similar effect sizes were not observed with DPP-4 Is or SGLT-2 Is (Fig. 2). Regarding acute myocardial infarction, the difference amounted to 22–26% for three (AMPLITUDE-O^16^, HARMONY Outcomes^13^, SUSTAIN-6^11^) out of eight studies^9–16^ . For stroke, the largest differences were 24–39% for three (REWIND^14^, PIONEER-6^15^, SUSTAIN-6^11^) out of eight studies^9–16^). Regarding cardiovascular death, the largest differences were 22–51% in GLP-1 RA studies (LEADER^10^, AMPLITUDE-O^16^, and PIONEER-6^15^), and the SGLT-2 I trial EMPA-REG Outcomes^1^). All-cause death was reduced by up to 22–49% (GLP-1 RAs: AMPLITUDE-O^16^ and PIONEER-6^15^; SGLT-2 I: EMPA-REG Outcomes^1^). For hospitalization because of congestive heart failure, a consistent 27–35% reduction was seen with all SGLT-2 Is^1–4^, but also with efpeglenatide (by 39%; AMPLITUDE-O^16^). Of note, not even all these large differences were significant (due to small numbers of events and sample sizes; Table 1 and Supplementary Table 3). Thus, for the majority of study results concerning the individual endpoints of interest, the power was not sufficient to detect the magnitude of differences as the have been described with respect to acute myocardial infarction, stroke, cardiovascular death, all-cause death, and hospitalization for congestive heart failure in the majority of studies, even for medications belonging to classes which in principle have proven the potential to reduce cardiovascular endpoints selected for the present analysis. The latter can be concluded from meta-analyses of cardiovascular outcomes trials examining SGLT-2 Is and GLP-1 RAs, which found significant effects on all endpoints, composite or individual, which have been explored in the present analysis, with the exception of stroke in trials employing SGLT-2 inhibitors^21–24^.

Based on the findings of our present analysis, we do not believe that it is justified to compare the effectiveness in reducing individual cardiovascular endpoints for the treatment with various compounds belonging to one class of glucose-lowering medications (e.g., within GLP-1 RAs), or even between different classes (e.g., comparing SGLT-2 Is and GLP-1 RAs). The main reason is that the power has not been found sufficient to detect typical event reductions observed in such trials, which mainly range from 10 to 15% (Fig. 1).

This is not to say that the studies which we have analysed are ill-powered for the purpose that they were designed for, e.g. the provision of evidence sufficient to support the claim of non-inferiority (safety) and with a chance to detect superiority, if the difference was, e.g., ≥ 15%, depending on the hypothesis guiding sample size calculation. Differences are obvious between studies aiming at the final proof of safety according to FDA criteria, and trials designed to satisfy criteria suggesting preliminary safety for getting a novel diabetes medication approved, however, with the commitment to perform a definitive post-marketing cardiovascular trial. The latter applies mainly to the two semaglutide trials, SUSTAIN-6^11^ (semaglutide s.c. once weekly) and PIONEER-6^15^ (oral semaglutide for once-daily administration). As mentioned in the results section, these trials in many respects lack a power that is sufficient to conclude superiority regarding several endpoints. As a result, non-significant differences sometimes had to be concluded even in the presence of impressive effect sizes (e.g., MACE in PIONEER-6^15^). Needless to say, a final determination of robustly estimated effect sizes is difficult under these circumstances. Similar considerations apply to AMPLITUDE-O (efpeglenatide) because of its relatively short duration^16^.

The results of cardiovascular outcomes trials show widely heterogeneous results based on the medication class employed, but also comparing results with glucose-lowering medications belonging to the same class (Figs. 1). This heterogeneity, most likely, is the result of differences which can be ascribed to the drugs themselves, but also to features related to the design, in- and exclusion criteria, patient characteristics (Supplementary Tables 1 and 2) and sample sizes (Table 1) of the clinical trials assessing clinical effects.

As can be derived from the effect sizes for the influence on various cardiovascular outcomes, there appears to be little heterogeneity with respect to SGLT- Is, in particular with respect to their effect on hospitalizations because of heart failure (Fig. 1)^1–4^. Likewise, there seems to be little heterogeneity with respect to differences between various DPP-4 inhibitors (with the potential exception of their influence on heart failure-related hospitalizations, Fig. 1). However, as has been previously noted, the class of GLP-1 RAs seems to be highly heterogeneous. In particular, lixisenatide has been almost without any hint for cardiovascular benefits (Fig. 1)^9^, which has been explained by the limited exposure to effective drug levels for a minor proportion of a 24 h period^27,28,33^. The development of other GLP-1 RAs also markedly differed with respect to pharmacokinetic properties and the scrutiny of defining effective, but tolerable doses^27,28^. These differences have proven relevant for the resulting effect sizes regarding glycaemic control and body weight reduction, which seem to be related to those reported for cardiovascular effects^27,28^, perhaps because both effects are related to optimized target engagement.

It is obvious from the present data, that smaller studies more likely yield erratic results (larger effect sizes compared to larger studies, which, nevertheless, often are not significant, or unexpectedly small effect sizes; Fig. 1). Therefore, when attempting to characterize cardiovascular properties of glucose-lowering medications, the emphasis should be on larger studies accruing sufficient numbers of events of interest. Along the same lines, larger, definite trial are underway to characterize cardiovascular effects of oral semaglutide in type 2 diabetes (SOUL trial, ClinicalTrials.gov: NCT03914326) and in populations characterized by obesity and related cardiovascular complications (SELECT trial, ClinicalTrials.gov: NCT03574597).

Our analysis has shown that cardiovascular outcomes studies performed as part of the development of novel glucose-lowering drugs are typically adequately powered for the main (primary) endpoint MACE, but that they have variable power, depending on sample size and the number of cardiovascular events accrued, to provide significance for the effect size of differences that are typically observed with SGLT-2 Is and GLP-1 RAs. As a result, indirect comparisons between placebo-controlled cardiovascular outcomes trials with different glucose-lowering drugs do not appear uniformly suitable to detect differences in their ability to elicit a unique pattern of effects on various cardiovascular endpoints.

## Materials and methods

### Study design

The present study is a systematic analysis of published cardiovascular outcomes trials examining glucose-lowering medications, belonging to the medication classes SGLT-2 Is^1–4^, DPP-4 Is^5–8^, and GLP-1 RAs^9–16^, in patients with type 2 diabetes and a high risk to develop cardiovascular complications, which used major adverse cardiovascular outcomes (“MACE”) as their primary endpoint, but also reported individual cardiovascular outcomes including MACE components (non-fatal acute myocardial infarction or stroke, cardiovascular death), all-cause death, and hospitalization for congestive heart failure. Studies reporting other populations (e.g., at high risk for renal endpoints (e.g., the CREDENCE trial^20^) or heart failure (DAPA HF trial^34^, EMPEROR reduced trail^35^), were not used for the present analysis. After a systematic literature search identifying suitable publications, data regarding the sample size (number of participants) and on cardiovascular outcomes (number of patients experiencing primary and secondary endpoints with active or placebo treatment (on a background of “standard of care”) were extracted. The power to detect 10, 15, 20 or 25% differences between active and placebo treatment was estimated. In addition, the power to detect a difference equivalent to that observed between active and placebo treatment in any particular study was also estimated.

### Literature search

The PubMed database was searched using Endnote 7.0. Search terms were lixisenatide, liraglutide, semaglutide, exenatide, albiglutide, dulaglutide, and efpeglenatide (GLP-1 RAs), saxagliptin, alogliptin, sitagliptin and linagliptin (DPP-4 Is), and empagliflozin, canagliflozin, dapagliflozin, and ertugliflozin (SGLT-2 Is) in the title and “major adverse cardiovascular outcomes” or MACE, myocardial infarction, stroke, cardiovascular death, all-cause death or total mortality, and hospitalization for (congestive) heart failure in the abstract. Retrieved literature was checked regarding suitability by analysing the abstracts, and—if found potentially suitable—the full manuscript. Reference lists of all retrieved publications were search for additional publications that could potentially be useful.

### Data extraction

Data from individual publications (including their online supplementary files) were transferred to a paper form systematically listing items of interest for the present analysis, independently by SB and MAN. Discrepancies were resolved by discussion with JJM. The main focus was on parameters characterizing the dimensions of the study with respect to participant numbers, and the number of primary and secondary outcomes and the resulting hazard ratios (with 95% confidence intervals) for MACE, acute myocardial infarction, stroke, cardiovascular death, all-cause death, hospitalization for heart failure reported with placebo and active treatment with SGLT-2 Is, DPP-4 Is, and GLP-1 RAs. In addition, patient characteristics at baseline were extracted in order to provide information on the selection of patient populations. These data were transferred to Excel spreadsheets for further use (e.g., for the generation of Tables and Figures).

Study quality was assessed applying the Jadad score^36^ and the Risk of Bias tool (https://www.riskofbias.info/)^37^. All publications turned out to be suitable for our analysis.

### Power calculation

Statistica 13.3 (TIBCO Software Inc. (2017). Statistica (software system for data analysis), version 13. http://statistica.io) was used to estimate the power of individual trials to detect differences by 10, 15, 20, or 25% vs. event rates published for placebo-treated patients. Input variables were the proportions of patients developing the endpoint of interest, and the total number of patients in this particular trial (sum of numbers treated with active drug and placebo). The output variable was the power estimated based on a log-rank test to compare survival curves between patients treated with active drug and placebo. A power of > 80% was tentatively defined as a meaningful prerequisite for drawing conclusions from significant differences.

### Statistics

Descriptive statistics use means ± standard deviations for continuous variables and numbers fulfilling certain criteria and the proportions (percentages) of the total population for categorical variables.

### Ethical approval and consent to participate

Not applicable, since the present study analyses published data on cardiovascular outcomes trials, not individual patients’ data. All the procedures were performed in accordance with the relevant guidelines and regulations.

## Supplementary Information

Supplementary Information.

## Data Availability

All data analysed and presented in the present manuscript have been taken from published manuscripts. No original data have been used.
