# Supplementary material for: Statistical power for MACE and individual secondary endpoints in cardiovascular outcomes trials for type 2 diabetes: a systematic review
Source: Sci Rep. 2022 Dec 6;12:21069. doi: 10.1038/s41598-022-25296-x (PMC9726861; doi:10.1038/s41598-022-25296-x)
Supplement: Supplementary file 1 — Supplementary Information. [file 41598_2022_25296_MOESM1_ESM.docx]

**Statistical power for MACE and individual secondary endpoints in cardiovascular outcomes trials for type 2 diabetes – a systematic review**

**Sebastian Birker^1^, Juris J. Meier^1,2^, Michael A. Nauck^1^**

Diabetes Division, Katholisches Klinikum Bochum, Ruhr-University Bochum, Bochum, Germany

Department of Internal Medicine, Augusta Hospital, Bochum, Germany

**Online Supplement**

**Supplementary Figure 1.** *Post hoc* power estimation of for cardiovascular outcomes concerning trials comparing novel glucose-lowering medications with placebo treatment (both on a background of standard of care) in patients with type 2 diabetes for all-cause death (upper row of panels) and hospitalization for congestive heart failure (lower row of panels). Left hand panels display the power calculated regarding the hazard ratio as reported in each individual study. The second through fourth columns of panels illustrate the power to detect differences by 10, 15, 20, or 25 % versus the proportion of patients with events reported with placebo treatment with SGLT-2 inhibitors (second column of panels), DPP-4 inhibitors (third column of panels) and GLP-1 receptor agonists (fourth column of panels). The power estimates for study results indicating a negligible difference to placebo treatment (≤ 10 %) are highlighted with a dashed rectangle, because small differences are typically associated with low power.

**Supplementary Figure 2.** Regression analyses relating confidence intervals for the hazard ratio (active treatment vs. placebo) for major adverse cardiovascular events (MACE) to parameters characterizing the study size (overall patient years of observation, upper left; number of MACE events, upper right; both reporting the sum of active plus placebo treatment; study duration, lower left; patient years of observation, lower right, both reporting the average of active and placebo treatment). The regression equations, the coefficient of correlation squared (r^2^), and the respective p-values for a significant association are also shown.

| **Supplementary Table 1.** Study characteristics (study population, patients completing study, vital status known and patient discontinuing study medication) | | | | | | | | | | | | | | | | |
| --- | --- | --- | --- | --- | --- | --- | --- | --- | --- | --- | --- | --- | --- | --- | --- | --- |
|  |  | Study population | | |  | Patients completing study | | |  | Vital status known | | |  | Patients discontinuing study medication | | |
| Class | Study acronym | total [n] | Placebo treatment [n] | Active drug treatment [n] |  | Placebo treatment [n] (proportion [%]) |  | Active drug treatment [n] (proportion [%]) |  | Placebo treatment [n] (proportion [%]) |  | Active drug treatment [n] (proportion [%]) |  | Placebo treatment [n] (proportion [%]) |  | Active drug treatment [n] (proportion [%]) |
| SGLT-2 inhibitors | EMPAREG-OUTCOME | 7020 | 2333 | 4687 |  | 2266 (97.1) |  | 4543 (96.9) |  | 2316 (99.3) |  | 4651 (99.2) |  | 683 (29.3) |  | 1097 (23.4) |
|  | CANVAS | 10142 | 4347 | 5795 |  | 4163 (95.8) |  | 5571 (96.1) |  | 4327 (99.5) |  | 5773 (99.6) |  | 1297 (29.9) |  | 1693 (29.2) |
|  | DECLARE-TIMI 58 | 17160 | 8578 | 8582 |  | 7854 (91.6) |  | 7936 (92.5) |  | 8532 (99.5) |  | 8546 (99.6) |  | 2151 (25.1) |  | 1811 (21.1) |
|  | VERTIS-CV | 8246 | 2747 | 5499 |  | 2389 (87.0) |  | 4824 (87.8) |  | 2730 (99.4) |  | 5457 (99.2) |  | 767 (27.9) |  | 1291 (23.5) |
| DPP4 inhibitors | SAVOR TIMI 53 | 16492 | 8212 | 8080 |  | 7998 (97.4) |  | 8078 (97.6) |  | 8140 (99.1) |  | 8205 (99.1) |  | 1705 (20.8) |  | 1527 (18.4) |
|  | EXAMINE | 5380 | 2679 | 2701 |  | 2361 (88.1) |  | 2403 (89.0) |  | 2663 (99.4) |  | 2692 (99.7) |  | 606 (22.6) |  | 564 (20.9) |
|  | TECOS | 14671 | 7339 | 7332 |  | 6905 (94.1) |  | 6972 (95.1) |  | 7123 (97.1) |  | 7180 (97.9) |  | 2008 (27.4) |  | 1901 (25.9) |
|  | CARMELINA | 6979 | 3485 | 3494 |  | 3430 (98.4) |  | 3458 (99.0) |  | 3485 (99.7) |  | 3494 (99.7) |  | 955 (27.8) |  | 834 (24.1) |
| GLP-1 receptor agonists | ELIXA | 6068 | 3034 | 3034 |  | 2924 (96.4) |  | 2929 (96.5) |  | 2992 (98.6) |  | 3005 (99.0) |  | 833 (27.5) |  | 727 (24.0) |
|  | LEADER | 9340 | 4672 | 4668 |  | 4513 (96.6) |  | 4529 (97.0) |  | 4655 (99.6) |  | 4656 (99.7) |  | n.r. |  | n.r |
|  | SUSTAIN-6 | 3297 | 1649 | 1648 |  | 1609 (97.6) |  | 1623 (98.5) |  | 1642 (99.6) |  | 1642 (99.6) |  | n.r. |  | n.r. |
|  | EXSCEL | 14752 | 7396 | 7356 |  | 7093 (95.9) |  | 7094 (96.4) |  | 7253 (98.1) |  | 7229 (98.3) |  | 3343 (45.2) |  | 3164 (43.0) |
|  | HARMONY OUTCOMES | 9463 | 4732 | 4731 |  | 4577 (96.7) |  | 4619 (97.6) |  | 4701 (99.3) |  | 4701 (99.4) |  | 1318 (27.9) |  | 1161 (24.5) |
|  | REWIND | 9901 | 4952 | 4949 |  | 4793 (96.8) |  | 4817 (97.3) |  | 4935 (99.7) |  | 4932 (99.7) |  | 310 (6.3) |  | 451 (9.1) |
|  | PIONEER 6 | 3183 | 1592 | 1591 |  | 1586 (99.6) |  | 1586 (99.7) |  | 1592 (100) |  | 1591 (100) |  | 155 (9.7) |  | 244 (15.3) |
|  | AMPLITUDE-O | 4076 | 1359 | 2717 |  | 1306 (96.1) |  | 2635 (97.0) |  | 1358 (99.9) |  | 2715 (99.9) |  | n.r. |  | n.r. |
| n.r.: not reported | | | | | | | | | | | | | | | | |

| **Supplementary Table 2.** Baseline patient characteristics | | | | | | | | | | | |
| --- | --- | --- | --- | --- | --- | --- | --- | --- | --- | --- | --- |
| Class | Study acronym | Age [years] | Female sex [n] (proportion [%]) | Body mass index [kg/m²] | Duration of diabetes [years] | HbA_1c_  [%] | HbA_1c_  [mmol/mol] | Blood pressure systolic/ diastolic) [mmHg] | LDL cholesterol [mg/dl] | Patients with established cardiovascular disease [n] (proportion [%]) | Patients with established heart failure [n] (proportion [%]) |
| SGLT-2 inhibitors | EMPAREG-OUTCOME | 63.1±8.6 | 2004 (28.5) | 30.6±5.3 | n.r. | 8.1±0.8 | 65±9 | 135/77 | 84.6±34.6 | 7020 (100) | 9706(10.1) |
|  | CANVAS | 63.3±8.3 | 3633 (35.8) | 32.0±5.9 | 13.5±7.8 | 8.2±0.9 | 66±10 | 137/78 | 88.5±34.6 | 6656 (65.6) | 1461 (14.4) |
|  | DECLARE-TIMI 58 | 63.8±6.8 | 6422 (37.4) | 32.1±6.0 | 11 * | 8.3±1.2 | 67±13 | 135/78 | 88.5±34.6 | 6974 (40.6) | 1724 (10.0) |
|  | VERTIS-CV | 64.4±8.1 | 2477 (30.0) | 31.9±5.4 | 12.9±8.3 | 8.2±1.0 | 66±11 | 133/77 | 89.1±38.2 | 8246 (100) | 1959 (23.8) |
| DPP4 inhibitors | SAVOR TIMI 53 | 65.0±8.6 | 5455 (33.1) | 31.1±5.6 | 11.9±8.9 | 8.0±1.4 | 64±15 | 137/79 | n.r. | 12959 (78.6) | 2105 (12.8) |
|  | EXAMINE | 60.9±10.0 | 1729 (32.1) | 28.7 * | 7.1 * | 8.0±1.1 | 64±12 | n.r. | 78.6±34.8 | 5380 (100) | 1501 (27.9) |
|  | TECOS | 65.5±8.0 | 4297 (29.3) | 30.2±5.7 | 11.6±8.1 | 7.2±0.5 | 55±5 | 135/77 | 91.0±57.8 | 10863 (74.0) | 2643 (18.0) |
|  | CARMELINA | 65.8±9.1 | 2589 (37.1) | 31.3±5.3 | 14.7±9.5 | 7.9±1.0 | 63±11 | 141/78 | 91.0±40.0 | 6911 (99.0) | 1873 (26.8) |
| GLP-1 receptor agonists | ELIXA | 60.3±9.7 | 1861 (30.7) | 30.2±5.7 | 9.3±8.3 | 7.7±1.3 | 61±15 | 130/n.r. | 78.5±35.3 | 6068 (100) | 1358 (22.4) |
|  | LEADER | 64.3±7.2 | 3337 (35.7) | 32.5±6.3 | 12.8±8.0 | 8.7±1.5 | 72±17 | 136/77 | 89.5±35.5 | 7598 (81.3) | 1305 (14.0) |
|  | SUSTAIN-6 | 64.6±7.4 | 1295 (39.3) | 32.8±6.2 | 13.9±8.1 | 8.7±1.5 | 72±17 | 136/77 | 82.3±45.6 | 2382 (72.2) ¶ | 777 (23.6) |
|  | EXSCEL | 61.9±9.4 | 5603 (38.0) | 31.8±5.9 | 12 * | 8.0±1.2 | 64±13 | 135/80 * | 88 * | 10782 (73.1) | 2389 (16.2) |
|  | HARMONY OUTCOMES | 64.1±8.7 | 2894 (30.6) | 32.3±5.9 | 14.2±8.8 | 8.7±1.5 | 72±17 | 135/77 | 81.7† | 9463 (100) | 1922 (20.3) |
|  | REWIND | 66.2±6.5 | 4589 (46.3) | 32.3±5.7 | 10.6±7.3 | 7.3±1.1 | 56±12 | 137/79 | 98.5±37.7 | 3114 (31.5) | 853 (8.6) |
|  | PIONEER 6 | 66.0±7.0 | 1007 (31.6) | 32.3±6.5 | 14.9±8.5 | 8.2±1.6 | 66±17 | 136/76 | 78.0^+^ | 2695 (84.7) | 388 (12.2) |
|  | AMPLITUDE-O | 64.5±8.2 | 1344 (33.0) | 32.7±6.2 | 15.4±8.8 | 8.9±1.5 | 74±17 | 135/7 | 79.6±37.7 | 3650 (89.6) | 737 (18.1) |
| Mean ± SD or [n] (proportion [%]); * only median reported; † only mean without SD reported; ¶ from *post hoc* analysis (Leiter et al. (1)) 76.8%; n.r.: not reported | | | | | | | | | | | |

| **Supplementary Table 3.** Sample size (total study population), median study duration, patient years of observation, and number of events reported with placebo or active drug treatment for each component (non-fatal myocardial infarction, non-fatal stroke an cardiovascular death) of major adverse cardiovascular events (MACE) | | | | | | | | | | | | | |
| --- | --- | --- | --- | --- | --- | --- | --- | --- | --- | --- | --- | --- | --- |
|  |  |  | Non fatal myocardial infarction | | |  | Non-fatal stroke | | |  | Cardiovascular deaths | | |
|  |  |  | Placebo treatment |  | Active drug treatment |  | Placebo treatment |  | Active drug treatment |  | Placebo treatment |  | Active drug treatment |
| Class | Study acronym |  | Events [n] (proportion [%]) |  | Events [n] (proportion [%]) |  | Events [n] (proportion [%]) |  | Events [n] (proportion [%]) |  | Events [n] (proportion [%]) |  | Events [n] (proportion [%]) |
| SGLT-2 inhibitors | EMPAREG-OUTCOME |  | 136 (5.8) |  | 251 (5.4) |  | 60 (2.6) |  | 150 (3.2) |  | 137 (5.9) |  | 172 (3.7) |
|  | CANVAS |  | 179 (4.1) |  | 200 (3.5) |  | 129 (3.0) |  | 146 (2.5) |  | 198 (4.5) |  | 239 (4.1) |
|  | DECLARE-TIMI 58 |  | 441 (5.1) |  | 393(4.6) |  | 231 (2.7) |  | 235 (2.7) |  | 249 (2.9) |  | 245 (2.9) |
|  | VERTIS-CV |  | 148 (5.4) |  | 310 (5.6) |  | 78 (2.8) |  | 157 (2.9) |  | 184 (6.7) |  | 341 (6.2) |
| DPP4 inhibitors | SAVOR TIMI 53 |  | 278 (3.4) |  | 265 (3.2) |  | 141 (1.7) |  | 157 (1.9) |  | 278 (3.4) |  | 269 (3.2) |
|  | EXAMINE |  | 173 (6.5) * |  | 187 (6.9) * |  | 32 (1.2) * |  | 29 (1.1) * |  | 130 (4.9) |  | 112 (4.1) |
|  | TECOS |  | 316 (4.3) |  | 300 (4.1) |  | 183 (2.5) |  | 178 (2.4) |  | 366 (5.0) |  | 380 (5.2) |
|  | CARMELINA |  | 135 (3.9) |  | 156 (4.5) |  | 73 (2.1) |  | 65 (1.9) |  | 264 (7.6) |  | 255 (7.3) |
| GLP-1 receptor agonists | ELIXA |  | 261 (8.6) |  | 270 (8.9) |  | 60 (2.0) |  | 67 (2.2) |  | 158 (5.2) |  | 156 (5.1) |
|  | LEADER |  | 317 (6.8) |  | 281 (6.0) |  | 177 (3.4) |  | 159 (3.8) |  | 278 (6.0) |  | 219 (4.7) |
|  | SUSTAIN-6 |  | 64 (3.9) |  | 47 (2.9) |  | 44 (2.7) |  | 27 (1.6) |  | 46 (2.8) |  | 44 (2.7) |
|  | EXSCEL |  | 480 (6.5) |  | 466 (6.3) |  | 193 (2.6) |  | 169 (2.3) |  | 383 (5.2) |  | 340 (4.6) |
|  | HARMONY OUTCOMES |  | 240 (5.0) |  | 181 (4.0) |  | 108 (2.0) |  | 94 (2.0) |  | 130 (3.0) |  | 122 (3.0) |
|  | REWIND |  | 212 (4.3) |  | 205 (4.1) |  | 175 (3.5) |  | 135 (2.7) |  | 346 (7.0) |  | 317 (6.4) |
|  | PIONEER 6 |  | 31 (1.9) |  | 37 (2.3) |  | 16 (1.0) |  | 12 (0.8) |  | 30 (1.9) |  | 15 (0.9) |
|  | AMPLITUDE-O |  | 53 (3.9) |  | 85 (3.1) |  | 25 (1.8) |  | 41 (1.5) |  | 50 (3.7) |  | 75 (2.8) |
| *: Myocardial infarction and stroke only reported as a component of MACE | | | | | | | | | | | | | |

**References quoted in the Online Supplement**

1. Leiter LA, Bain SC, Hramiak I, Jodar E, Madsbad S, Gondolf T, Hansen T, Holst I, Lingvay I: Cardiovascular risk reduction with once-weekly semaglutide in subjects with type 2 diabetes: a post hoc analysis of gender, age, and baseline CV risk profile in the SUSTAIN 6 trial. Cardiovasc Diabetol 2019;18:73
